# Emergency surgery for hiatus hernias: does technique affect outcomes? A single-centre experience

**DOI:** 10.1007/s13304-023-01482-y

**Published:** 2023-03-03

**Authors:** Mohamed Alasmar, Iona McKechnie, Ramakrishna P. C. Chaparala

**Affiliations:** 1 Department of Oesophago-Gastric Surgery, Salford Royal NHS Foundation Trust, Salford Royal Hospital, Stott Lane, Salford, M6 8HD UK; 2 Division of Cancer Sciences, School of Medical Sciences, Faculty of Biology, Medicine and Health, University of Manchester, Manchester, UK

**Keywords:** Hiatal hernia, Fundoplication, Gastropexy, Emergency surgical procedure, Stomach, Recurrence risk

## Abstract

**Background:**

Emergency surgery for a hiatus hernia is usually a high-risk procedure in acutely unwell patients. Common surgical techniques include reduction of the hernia, cruropexy then either fundoplication or gastropexy with a gastrostomy. This is an observational study in a tertiary referral centre for complicated hiatus hernias to compare recurrence rates between these two techniques.

**Methods:**

Eighty patients are included in this study, from October 2012 to November 2020. This is a retrospective review and analysis of their management and follow-up. Recurrence of the hiatus hernia that mandates surgical repair was the primary outcome of this study. Secondary outcomes include morbidity and mortality.

**Results:**

In total, 38% of the patients included in the study had fundoplication procedures, 53% had gastropexy, 6% had complete or partial resection of the stomach, 3% had fundoplication and gastropexy and one patient had neither (*n* = 30, 42, 5, 2,1, respectively). Eight patients had symptomatic recurrence of the hernia which required surgical repair. Three of these patients had acute recurrence and 5 after discharge. 50% had undergone fundoplication, 38% underwent gastropexy and 13% underwent a resection (*n* = 4, 3, 1) (*p* value = 0.5). 38% of patient had no complications and 30-day mortality was 7.5%

**Conclusion:**

To our knowledge, this is the largest single centre review of outcomes following emergency hiatus hernia repairs. Our results show that either fundoplication or gastropexy can be used safely to reduce the risk of recurrence in the emergency setting. Therefore, surgical technique can be tailored based on the patient characteristics and surgeon experience, without compromising the risk of recurrence or post-operative complications. Mortality and morbidity rates were in keeping with previous studies, which is lower than historically documented, with respiratory complications most prevalent. This study shows that emergency repair of hiatus hernias is a safe operation which is often a lifesaving procedure in elderly comorbid patients.

## Introduction

Hiatal hernias (HH) typically occur in the older population, when the gastroesophageal junction and fundus of the stomach have herniated above the diaphragm. The biggest concern is the risk of volvulus of the stomach in the chest, resulting in obstruction, strangulation and subsequent necrosis of the stomach wall. If this happens, patients will present acutely, and very unwell, with Borchardt’s triad of symptoms—severe epigastric pain, intractable retching and inability to pass a nasogastric tube. Patients often have other symptoms such as dysphagia, chest pain, reflux, respiratory distress and sepsis.

If a patient is suitable for surgery, the aim of a surgical procedure is to restore the stomach below the diaphragm to relieve the obstruction and restore blood flow. Necrotic areas can be resected, and steps taken to reduce the risk of recurrence of volvulus. Historically, this emergency procedure was reported as having a high mortality and morbidity rate, with case series reporting up to 40% mortality. However, with the development of surgical techniques, more recent papers have shown a reduced mortality rate of 2.7–8% [1–4].

Various techniques are described for the emergency surgical management of HH. For very unstable patients, an endoscopic reduction can be done [5]. If more stable, a laparoscopic or open repair can be done. The aim of the procedure is to reduce the hernial contents, to relieve the acute obstruction, and then reduce the risk of recurrence by dissecting the hernia sac, reapproximating the crura and anchoring the stomach below the diaphragm. If there is any necrosis of the stomach wall, a partial or complete resection is required. There are various surgical techniques that can be used, including fundoplication and gastropexy. There are limited data comparing the outcomes of different techniques in emergency HH repairs. This study aimed to identify surgical techniques used, and compare outcomes in recurrence, mortality and post-operative complications.

## Materials and methods

In a single tertiary gastroesophageal centre in Greater Manchester, a retrospective observational study was done. Consecutive patients who had emergency hiatal hernia repairs were identified, and data collected from their electronic patient records. Patients were identified from emergency operation records, and their medical records and imaging reviewed. Written patient consent was not applicable to this study. Local audit department approval was obtained. Exclusion criteria were patients with traumatic diaphragmatic hernias, and patients with previous oesophagectomies or gastrectomies. These results are being published in a separate study.

80 patients were identified from October 2012 to November 2020. Patients were either transferred from other hospitals or admitted directly to the tertiary centre. There were multiple surgeons who operated in the unit, and surgical technique was not standardised. The surgical technique used was based on the individual surgeons experience and patient characteristics.

Data were collected using Excel, and included patient demographics, clinical presentation, investigation results, operative procedure and post-operative care and follow-up. The primary outcome was symptomatic recurrence of hiatus hernia requiring revisional surgery. Secondary outcomes were post-operative mortality, and post-operative complications.

Descriptive statistics were used to report participant characteristics and outcomes in this study, including mean, standard deviation and 95% confidence intervals. Statistical analyses of the present study were performed using the R statistical package (R Foundation for Statistical Computing, Vienna, Austria. https://www.R-project.org/). Shapiro test was used to test for normality of continuous variables, the Wilcoxon test to compare nonparametric continuous variables, Pearson's chi-squared test and Fisher's exact test for categorical variables, logistic regression used to adjust for comorbidities and the log-rank test for survival analysis.

## Results

Eighty patients were identified who had emergency surgery for hiatal hernias over the time period. All patients presenting to any emergency department in Greater Manchester with acute deterioration secondary to HH were referred to the tertiary unit, and those requiring emergency surgery were transferred for operative management. Therefore, this study represents the whole population of Greater Manchester.

The median age was 71 years (range 22–93), of whom 35 were male, and 45 female. 70% of patients (*n* = 56) were transferred from other hospitals to the tertiary unit.

The majority of the patients (*n* = 55, 69%) were known to have a hiatus hernia prior to this admission. 45 (56%) of these patients were previously known to their local upper gastrointestinal surgical department, with 4 awaiting elective repair. The other 41 (51%) patients had not been offered elective repairs and were being managed using a watchful waiting approach. 13% (*n* = 10) patients had previously had some form of hiatal or anti-reflux surgery.

As shown in Table 1, the most common presentation symptoms were vomiting, abdominal pain and reflux.

**Table 1:** The most common presenting symptoms of emergency hiatus hernias

Symptom	Frequency
Dysphagia	19
Regurgitation	18
Reflux/heartburn	21
Vomiting	62
Shortness of breath	16
Sepsis	9
Chest pain	11
Abdominal pain	60

All patients had cross-sectional imaging prior to operative intervention. 25% (*n* = 20) patients had endoscopic studies, and 14% (*n* =11) had dynamic fluoroscopy. These were done to aid diagnosis and surgical planning.

86% (*n* = 69) patients had laparoscopic procedures, with 11% (*n* = 9) requiring subsequent conversion to open. 14% (*n* = 11) patients had open surgery. Intraoperative findings included organ ischaemia in 11%, perforation in 10%, mediastinal contamination in 4%, and 14% patients had other abdominal organs herniated into the chest (*n* = 9,8,3,11). 7 patients (9%) had a partial or complete gastrectomy due to necrosis.

There were a variety of surgical techniques used. These were classified as either fundoplication, gastropexy combined with a gastrostomy, both fundoplication and gastropexy, gastric resection, or none of the above. Fundoplication was either using a Watson Anterior 180 degree wrap, Toupet 270-degree posterior wrap, or a Nissen’s 360 degree wrap. Technique used depended on the surgeon’s preference. Gastropexy was done using 3 sutures to anchor the stomach below the left hemidiaphragm. This was combined with a gastrostomy, using a percutaneous endoscopic gastrostomy (PEG) or Foley catheter placed proximally to the pylorus to further anchor the stomach to the anterior abdominal wall. 38% had a fundoplication procedure, 53% had a gastropexy and gastrostomy and 3% had both a fundoplication and gastropexy. 6% had a complete gastrectomy due to necrosis, and 1 patient (1%) had only a laparoscopic reduction, due to intra-operative instability. This is summarised in Table 2*.*

**Table 2:** Frequency of the procedures performed

Fundoplication	30
Gastropexy +/- Gastrotomy	42
Fundoplication and Gastropexy	2
Resection	5
None	1
Total	80

The median length of stay post-surgery was 8 days. All patients were followed up in clinic after discharge.

### Rates of recurrence

8 patients (10%) had a symptomatic recurrence of their hiatus hernia requiring further surgery. 3 of these patients had acute recurrence post-operatively and required further emergency surgery to re-reduce the hernia. The other 5 patients had symptomatic recurrence later which was managed electively, with the earliest representing 9 months post-operatively.

Table 3 shows the operative techniques used for the patients who had recurrence. Statistical analysis gave a *p* value of 0.5, suggesting that the surgical technique used does not affect the rate of recurrence in emergency surgery. There was not statistically significant difference when we have adjusted recurrence rates to comorbidities using Charlson comorbidity score.

**Table 3:** Number of recurrences in each procedure

	In-hospital recurrence	Post-discharge recurrence	Total
Fundoplication	2	2	4
Gastropexy	1	2	3
Fundoplication and gastropexy	0	0	0
Resection	0	1	1
None	0	0	0
Total	3	5	8
*p* value	0.6856	0.5072	0.4977

### Mortality and morbidity

38% of patients had no complications during their admission. 30-Day post-operative mortality was 7.5% with 6 patients dying within 30 days of their procedure. The most frequently occurring complications were respiratory, with pneumonia being the most common (Table 4). The rate of most complications showed no statistical difference between techniques.

**Table 4:** Details of complications for each procedure

	Total	Fundoplication	Gastropexy	Fundoplication and gastropexy	Resection	None	*P* value
Total number of cases/*n*	80	30	42	2	5	1	
Gastrointestinal							
Ileus	8	1	6	1	0	0	0.1767
GI bleeding required transfusion/intervention`	2	1	1	0	0	0	1
Liver dysfunction	2	0	2	0	0	0	0.6013
Leak	5	1	2	0	2	0	0.1685
Respiratory							
Pneumonia	27	7	15	0	4	1	0.0383
Pleural effusion required drainage	6	1	3	0	2	0	0.1196
Pneumothorax required treatment	2	2	0	0	0	0	0.3288
Atelectasis mucus plugging requiring bronchoscopy	3	2	0	1	0	0	0.05158
Respiratory failure requiring reintubation	5	2	1	1	1	0	0.08094
Acute Aspiration	7	1	4	1	1	0	0.1516
Tracheostomy	2	2	0	0	0	0	0.3346
Cardiac							
Cardiac arrest requiring CPR	2	0	2	0	0	0	0.6013
MI	1	0	0	0	0	1	0.0125
Atrial dysrhythmia requiring treatment	20	5	12	0	2	1	0.2424
Ventricular dysrhythmia requiring treatment	2	1	1	0	0	0	1
CHF requiring treatment	9	2	5	0	1	1	0.154
Infection							
SSI	4	0	4	0	0	0	0.356
Acute abdominal wall dehiscence	3	1	2	0	0	0	1
Wound infection requiring opening or antibiotics	6	1	5	0	0	0	0.6829
Central IV line infection requiring removal or antibiotics	3	0	2	0	1	0	0.2468
Intrathoracic abscess	4	0	2	0	2	0	0.03961
Intraabdominal abscess	3	1	2	0	0	0	1
Generalised sepsis	7	2	4	0	1	0	0.6414
Other infections requiring antibiotics	22	6	12	0	3	1	0.1373
Renal							
AKI	8	2	4	0	1	1	0.129
AKI requiring dialysis/CVVH	2	0	1	0	0	1	0.03165
UTI	9	2	7	0	0	0	0.5982
Urinary retention requiring reinsertion of catheter, delayed discharge or discharge with catheter	3	0	3	0	0	0	0.4632
Thrombosis							
DVT	1	0	1	0	0	0	1
PE	1	0	1	0	0	0	1
Peripheral thrombophlebitis	1	0	1	0	0	0	1

## Discussion

To our knowledge, this is the largest single-centre retrospective study reviewing emergency HH repairs, surgical techniques used and the outcomes.

### Timing of surgery

It has been reported that up to 50% of the general population have a hiatus hernia [6]. The majority of these are sliding hiatus hernias, with minimal risk of complications. 5–10% have paraoesophageal hernias, which can be divided into Types II, III and IV depending on the degree of herniation. These patients are at an increased risk of serious complications, including gastric volvulus causing gastric outlet obstruction, strangulation, gastric wall necrosis and perforation. It is difficult to predict how many patients with HH will develop these complications. An elective repair of HH was traditionally advocated to reduce this risk. The procedure would involve reducing the hernia, as well as securing the stomach below the diaphragm to reduce the risk of recurrence. This typically results in an average inpatient stay of 4.9 days and post-operative mortality of 1.2% [7].

However, more recent studies have shown the risk of acute progression requiring emergency surgery is less than previously thought and could be as low as 1.1% [8]. Therefore, the peri-operative mortality of an elective repair is comparable to the risks of developing acute progression of a HH. It is estimated an elective surgical repair would benefit less than 20% of patients with mild symptoms [9]. As a result of this, watchful waiting is now frequently advised in patients who are asymptomatic or only have mild symptoms. In symptomatic patients, an elective repair is recommended [10]. In this series, 51% patients were being managed on the watchful waiting pathway, and 5% were awaiting elective repair (*n* = 41, 4). It would be interesting to compare this data to the total number of patients on the watchful waiting pathway to further understand the rate of acute deterioration. 49% of patients were not known to have a hiatal hernia prior to admission. It is typically difficult to identify patients with hiatal hernias due to the vague symptoms that develop over a long period of time. It is not known whether these patients were symptomatic prior to their acute presentation. It is possible that some of these patients may have benefitted from an elective repair if their condition had been identified earlier.

### Surgical technique

There is ongoing debate about surgical techniques used to reduce the risk of recurrence of hiatus hernia in the acute setting. Common techniques used include fundoplication, gastropexy alone, gastropexy with a gastrostomy or a combination of these. In this study, recurrence was defined as a patient becoming symptomatic requiring another procedure. Many papers investigate for radiological recurrence but it has been shown that radiological recurrence rarely correlates with recurrence of symptoms [11]. Therefore, in keeping with other recent studies, this study focused on symptomatic recurrence, as this is clinically relevant to both patient and surgeon [12].

There is ongoing discussion about the use of fundoplication in both the elective and emergency setting for HH repairs. Some consider fundoplication as a routine step in elective HH repairs. This is recommended in the SAGE guidelines [13] and is used in most studies on elective repairs [6, 14–18]. In patients with preoperative reflux, a fundoplication can both objectively and subjectively reduce their symptoms post-operatively [19]. It has also been shown that some patients without preoperative reflux can become symptomatic after a HH repair. It is thought that surgical reduction of the hernia compromises the natural anti-reflux system, inducing new reflux symptoms. Therefore some studies recommend the use of fundoplication in all elective patients for HH repair [6, 17]. However, fundoplication is not without risks, especially post-operative dysphagia and migration of the wrap [20].

In an emergency presentation, the benefit of fundoplication is less understood. It is suggested that a fundoplication increases the bulk of the gastro-oesophageal junction, and therefore anchors it below the diaphragm reducing the risk of recurrence of herniation [16, 17, 21]. However, there is no clear data to support this [22, 23]. In patients with symptomatic reflux, a fundoplication will help their symptoms, but performing a fundoplication increases the time and complexity of the emergency procedure. It is therefore only advisable to attempt a fundoplication in an emergency case when the patient is stable intra-operatively and it is safe to have longer time under anaesthetic [24].

Another well-published technique to reduce the risk of recurrence, is the use of a gastropexy, often combined with a gastrostomy. By securing the reduced stomach to the anterior abdominal wall with sutures and a gastrostomy tube, the risk of reherniation is reduced. This technique was first described by Boerema in 1969 [25]. Studies have shown that the use of a gastropexy can reduce the risk of recurrence, with one study showing the inclusion of a gastropexy reduces the risk of recurrence to 0% [26–28]. The biggest advantage of this technique is the relative speed, providing a fast but simple way of securing the acutely herniated and volved stomach. This is particularly useful in acutely unwell patients who are unstable intraoperatively. The addition of a gastrostomy using either a PEG or Foley catheter can also be used post-operatively to decompress the stomach and improve patient comfort [29].

In this series, both fundoplication and gastropexy with gastrostomy were used, and in 2 patients both techniques were used combined, as shown in Table 2. The technique used depended on the views and experiences of the surgeon as well as the perioperative stability of the patient. Statistical analysis showed that there was no difference in the rates of recurrence, either acutely or long term, between the two techniques (*p* = 0.5). This shows that either technique can be used without compromising the risk of recurrence. Therefore, the technique used can be tailored to the specific patient. For example, if they have symptomatic reflux a fundoplication can be used to manage this. If their stomach is particularly distended and there is a risk of an atonic stomach post-operatively, a gastropexy using a gastrostomy may be beneficial to deflate the stomach. If the patient is unstable intraoperatively, a gastropexy may be more appropriate given its speed compared to a fundoplication. Either technique can be used, with similar effects in reducing the risk of recurrence.

Two patients had both a gastropexy and fundoplication. Neither of them had recurrence requiring a further procedure. Further research would be beneficial to understand if the combination of procedures generates greater benefits than when used alone.

### Mortality and morbidity

In this study, mortality was 7.5% (*n* = 6) within 30 days of index procedure, which is in keeping with other studies [2–4]. 2 patients developed fatal aspiration pneumonia, 1 patient with known cardiac issues had a cardiac arrest, 1 patient developed multiorgan failure secondary to sepsis, 1 patient caught COVID during their admission, and 1 patient developed peritonitis, likely from gastric perforation. 1 patient remained an inpatient for 6 months due to a slow healing oesophageal leak, and then developed COVID and died. This was not counted in the 30-day mortality rate. Factors which have been shown to increase mortality are age > 70 and a Charlson Comorbidity Index (CCI) of greater than 3. 53% (*n* = 42) patients in this study were over the age of 70, and 44% (*n* = 35) patients had a CCI > 3.

As seen in other studies, morbidity was not as high as expected for a major emergency procedure [8]. Overall, 39% of patients did not suffer any complications post-operatively. Of those that did have complications, many of them were minor complications requiring minimal intervention and not affecting the length of stay (33% Clavien–Dindo scale I and II). 11% of patients required some form of radiological intervention, most frequently for pleural effusions or mediastinal collections requiring drainage. 4 patients required a return to theatre—2 for acute recurrence of HH, 1 for perforation from gastric necrosis, and 1 for management of an oesophagojejunal leak that developed post-total gastrectomy.

Pneumonia was the most commonly occurring complication. This is likely due to the nature of the pathology, with respiratory compromise due to the HH, recurrent vomiting increasing the chances of aspiration, thoracic reduction of the HH and a prolonged general anaesthetic for surgery. This has also been noted in other studies, reflecting the comorbid nature of these patients [1, 30].

Further analysis of the rates of complications between the different surgical techniques was done. This showed that for most recorded complications, there was no statistical significance in complication rates for the different techniques. There was a statistically significant difference in the rates of post-operative pneumonia when comparing patients who had gastropexy to fundoplication (*p* = 0.03, RR 1.53). It is likely that this increased risk is confounded by the more unstable pre-operative state of patients who had a gastropexy, rather than the surgical technique used. As they were more unwell on admission, they were more likely to have a gastropexy (shorter, less complex procedure) than a fundoplication, but were also more comorbid (higher CCI, 3.1 v 3.6), and likely to develop a post-operative complication such as pneumonia. Other complications that showed a significant difference in rates between techniques were myocardial infarction, acute kidney injury requiring dialysis and intra-thoracic abscesses. However, the rates were so low that a larger study would be needed to show these were truly statistically significant.

Overall, the mortality and morbidity was similar to that seen in other studies, and not as high as previously thought [2, 11].

### Limitations

There are some limitations of this study which require further discussion. The study design as a retrospective observational study is not the most appropriate design to compare two operative techniques. However, designing a randomised control trial to look at the differences between these techniques would be very difficult because of the rarity of the pathology and the urgency of surgery. We hope this may be possible in the future, with the centralisation of oesophagogastric services in this hospital. In this study, we present the best available evidence in relation to techniques of managing emergency hiatus hernias.

This study could not adjust and account for patients’ sociodemographic characteristics and co-morbidities beyond those discussed above. This was not possible due to the small numbers of the cases included in the study. Future studies done over a longer time period, and therefore, with more data would allow further analysis to understand the implications of these.

## Conclusion

To our knowledge, this is the largest single-centre retrospective review of outcomes following emergency hiatus hernia repairs. Our results show that either fundoplication or gastropexy can be used safely to reduce the risk of recurrence in the emergency setting. Therefore, surgical technique can be tailored based on the patient characteristics and surgeon experience, without compromising the risk of recurrence or post-operative complications. Mortality and morbidity rates were in keeping with previous studies, which is lower than historically documented, with respiratory complications most prevalent. This study shows that emergency repair of HH is a safe operation which is often a lifesaving procedure in elderly comorbid patients.

## Declarations

### Human and animal participants

Written patient consent did not apply to this study. as highlighted in the methods section
